# Effects of Remimazolam, Propofol, and Sevoflurane on Cerebral Oxygenation During Gynecologic Laparoscopic Surgery: A Prospective Randomized Study

**DOI:** 10.7759/cureus.111674

**Published:** 2026-06-28

**Authors:** Woojin Nam, Jihyun An, Kyeonghyo Kim, Yumin Kim

**Affiliations:** 1 Department of Anesthesiology and Pain Medicine, Daegu Fatima Hospital, Daegu, KOR

**Keywords:** near-infrared spectroscopy, propofol, regional cerebral oxygen saturation, remimazolam, sevoflurane, trendelenburg position

## Abstract

Background and aim: Laparoscopic gynecologic surgery frequently requires Trendelenburg positioning and carbon dioxide insufflation, both of which may alter cerebral oxygen dynamics. While the effects of propofol and sevoflurane on regional cerebral oxygen saturation have been investigated previously, limited information is available regarding remimazolam. This study aimed to compare changes in rSO₂ among patients receiving remimazolam, sevoflurane, or propofol anesthesia during gynecologic laparoscopic surgery in the Trendelenburg position. The primary endpoint was the change in rSO₂ measured at predefined perioperative time points among the three anesthetic groups.

Methods: A total of 72 women with American Society of Anesthesiologists (ASA) physical status I-II who were scheduled for elective gynecologic laparoscopic surgery were included in this prospective randomized study. Participants were allocated equally to maintenance anesthesia with remimazolam (R group), propofol (P group), or sevoflurane (S group). Regional cerebral oxygen saturation was continuously assessed using near-infrared spectroscopy. Regional cerebral oxygen saturation (rSO₂) values were recorded before anesthetic induction, 5 min after induction, 5 min following Trendelenburg positioning, and 5 min after restoration of the neutral position. Temporal changes in rSO₂ and between-group differences were analyzed using repeated-measures ANOVA.

Results: Baseline demographic and perioperative characteristics were comparable among groups. Regional cerebral oxygen saturation increased after induction and remained preserved throughout surgery in all groups. Repeated-measures ANOVA demonstrated a significant effect of time on rSO₂ (F {3, 207} = 32.89, p < 0.001) and a significant group-by-time interaction (F {6, 207} = 3.58, p = 0.002), whereas the overall group effect was not significant (F {2, 69} = 1.71, p = 0.189). Although the sevoflurane group showed numerically higher rSO₂ values, no significant overall difference in cerebral oxygenation was observed among groups. Bispectral index (BIS) values were significantly higher in the remimazolam group than in the propofol and sevoflurane groups (F {2, 69} = 45.81, p < 0.001). Mean arterial pressure after return to the neutral position was significantly higher in the remimazolam group than in the propofol and sevoflurane groups (p = 0.003). Cerebral oxygen desaturation occurred in one patient in the propofol group and in no patients in the remimazolam or sevoflurane groups.

Conclusions: Remimazolam, propofol, and sevoflurane all maintained adequate cerebral oxygenation during gynecologic laparoscopic surgery performed in the Trendelenburg position. Although sevoflurane demonstrated numerically higher rSO₂ values, no significant overall difference in cerebral oxygenation was identified among anesthetic techniques. Remimazolam provided cerebral oxygenation comparable to that of propofol and sevoflurane while offering superior hemodynamic stability, suggesting that it may be a useful alternative anesthetic agent for procedures requiring Trendelenburg positioning.

## Introduction

Near-infrared spectroscopy (NIRS) is widely used as a noninvasive monitoring modality for assessing regional cerebral oxygenation during anesthesia and surgery. Regional cerebral oxygen saturation (rSO₂) reflects the balance between cerebral oxygen delivery and consumption and may be influenced by several physiological factors, including cerebral perfusion, arterial oxygen content, hemoglobin concentration, and the cerebral metabolic rate of oxygen (CMRO₂) [1]. Because NIRS provides continuous real-time information regarding cerebral oxygen status, it has been increasingly incorporated into perioperative neurological monitoring. Previous studies have demonstrated its usefulness in detecting cerebral hypoperfusion and ischemic events during carotid endarterectomy [2]. However, studies evaluating cerebral oxygenation during gynecologic laparoscopic surgery performed in the Trendelenburg position have yielded inconsistent findings. Some investigators reported reductions in rSO₂ during surgery [3], whereas others observed increased rSO₂ values under comparable operative conditions [4].

Volatile anesthetics such as sevoflurane generally reduce CMRO₂ at low concentrations, which may induce cerebral vasoconstriction; however, at higher concentrations, their direct cerebral vasodilatory effects become dominant, increasing cerebral blood flow (CBF) and cerebral blood volume (CBV), potentially leading to increased intracranial pressure (ICP). In contrast, propofol dose-dependently reduces CMRO₂, CBF, CBV, and ICP [5,6]. Accordingly, propofol exerts a greater suppressive effect on cerebral blood flow than sevoflurane. Previous studies have suggested that sevoflurane anesthesia may better preserve rSO₂ than propofol anesthesia during laparoscopic surgery performed in the Trendelenburg position [7].

Remimazolam is a novel ultra-short-acting benzodiazepine [8]. Although it has a pharmacological profile similar to that of conventional benzodiazepines such as midazolam, it undergoes rapid organ-independent metabolism to an inactive metabolite and is therefore unlikely to cause prolonged sedative effects [2]. Compared with propofol, remimazolam is associated with less injection pain and fewer cardiovascular and respiratory depressant effects [9]. Similar to other benzodiazepines, remimazolam reduces cerebral metabolic activity; however, its effects on cerebral oxygenation during surgery remain poorly understood. Furthermore, limited evidence exists regarding its influence on cerebral oxygenation under conditions associated with altered cerebral hemodynamics, such as pneumoperitoneum and the Trendelenburg position. Unlike propofol and volatile anesthetics, the effects of remimazolam on cerebral oxygenation and cerebral hemodynamics during laparoscopic surgery in the Trendelenburg position have not been well characterized.

## Materials and methods

Patient selection

This prospective randomized trial included women scheduled for elective gynecologic laparoscopic procedures under general anesthesia. Eligible participants were between 20 and 65 years of age and had an American Society of Anesthesiologists (ASA) physical status of I or II. A total of 72 patients were recruited for the study.

Patients were excluded if they had a history of cerebrovascular disease, uncontrolled hypertension, obesity (BMI ≥ 25 kg/m²), known hypersensitivity to study medications, drug or alcohol misuse, or uncontrolled anemia. Before participation, all patients received detailed information regarding the study protocol and anesthetic management and provided written informed consent. Eligibility was determined without consideration of race or socioeconomic background.

Randomization and blinding

Following study enrollment, participants were distributed equally among three treatment groups according to the anesthetic used for maintenance of anesthesia. Patients receiving sevoflurane, propofol, or remimazolam were assigned to the S, P, and R groups, respectively.

Group assignments were generated using a computer-generated randomization sequence before study initiation and concealed within sequentially numbered opaque envelopes. A total of 72 participants underwent randomization, with 24 patients allocated to each study group.

Because the anesthetic techniques differed among groups, blinding of the attending anesthesiologist was not feasible. Nevertheless, study outcomes were evaluated using predefined objective measures, and statistical analyses were performed after completion of data collection.

Sample size consideration

The sample size was determined with reference to previous studies evaluating regional cerebral oxygen saturation during gynecologic laparoscopic surgery in the Trendelenburg position. Based on the sample sizes used in comparable investigations and the feasibility of patient recruitment during the study period, 72 patients were enrolled and equally allocated to the three study groups (24 patients per group).

Anesthetic management

Thirty minutes before surgery, all patients received intramuscular glycopyrrolate 0.2 mg and midazolam 2 mg, together with intravenous famotidine 20 mg as premedication. Upon arrival in the operating room, standard monitoring, including electrocardiography (ECG), pulse oximetry (SpO₂), noninvasive blood pressure (NIBP), and bispectral index (BIS) monitoring, was applied. rSO₂ was continuously monitored using a near-infrared spectroscopy cerebral oximeter (INVOS 5100C; Dublin, Ireland: Medtronic). Sensors were placed bilaterally on the forehead above the eyebrows, and the mean value of both sides was used for analysis. After application of all monitoring devices, patients underwent 2 min of preoxygenation before induction.

Induction of anesthesia was performed according to group allocation. Patients in the S group received propofol 2 mg/kg, whereas those in the P group received propofol via target-controlled infusion with an effect-site concentration target of 4 μg/mL. In the R group, remimazolam was administered at 6 mg/kg/h until loss of consciousness was achieved. Remifentanil was delivered in all groups using target-controlled infusion with an effect-site concentration target of 2 ng/mL.

After unconsciousness had been established and BIS values reached the target range of 40-65, neuromuscular blockade was achieved with rocuronium 0.6 mg/kg, followed by tracheal intubation. Mechanical ventilation was maintained using an oxygen-air mixture with FiO₂ of 0.5, while end-tidal carbon dioxide (EtCO₂) was maintained between 35 and 40 mmHg.

During the procedure, anesthetic administration was adjusted to maintain BIS values within the target range of 40-65. Sevoflurane concentration was adjusted between 1.5 and 2.0 vol% in the S group. Propofol was maintained at an effect-site concentration of 2.5-4.0 μg/mL in the P group. Remimazolam was maintained at 1-2 mg/kg/h in the R group.

Mean arterial pressure was maintained within 30% of baseline values. Hypotension (MAP: < 60 mmHg) or bradycardia (HR < 45 beats/min for > 5 min) was treated with phenylephrine 0.1 mg, ephedrine 4 mg, or atropine 0.5 mg as clinically indicated. Hypertension (MAP > 110 mmHg for > 5 min) was treated with nicardipine 1 mg.

After induction, patients were placed in the lithotomy and Trendelenburg positions. Pneumoperitoneum was established using carbon dioxide insufflation. At the completion of surgery, patients were returned to the neutral position. At the end of surgery, all anesthetic agents and remifentanil infusions were discontinued. Neuromuscular blockade was reversed with sugammadex 2 mg/kg intravenously. Patients were transferred to the postanesthesia care unit (PACU) after confirmation of adequate recovery.

Outcome measures

The primary outcome was the change in rSO₂ among the three anesthetic groups. Measurements of rSO₂, noninvasive blood pressure, heart rate, SpO₂, EtCO₂, and esophageal temperature were obtained at the following three predefined perioperative time points: 5 min after induction of anesthesia (T1), 5 min after placement in the Trendelenburg position (T2), and 5 min after return to the neutral position (T3). For analysis, the mean value of the bilateral rSO₂ measurements was used.

Cerebral oxygen desaturation was defined as either a decrease in rSO₂ greater than 20% from baseline or an absolute rSO₂ value below 40 on either side. Secondary outcomes included the incidence of cerebral oxygen desaturation, intraoperative hemodynamic variables, total remifentanil consumption, duration of surgery and anesthesia, estimated blood loss, intraoperative fluid administration, and postoperative adverse events, including nausea, vomiting, and neurological complications.

Statistical analysis

Continuous variables are presented as mean ± standard deviation (SD), whereas categorical variables are reported as frequencies and percentages. Baseline demographic and perioperative characteristics were compared according to data type. Continuous variables were analyzed using one-way analysis of variance (ANOVA), while categorical variables were evaluated using either the chi-square test or Fisher’s exact test, as appropriate.

Longitudinal changes in rSO₂, bispectral index (BIS), mean arterial pressure (MAP), and heart rate (HR) were assessed using repeated-measures ANOVA. The effects of study group, measurement time, and group-by-time interaction were examined. When significant overall group differences were identified, post hoc pairwise comparisons were performed using the Bonferroni correction to account for multiple testing. All statistical tests were two-sided, and a p < 0.05 was considered statistically significant. Statistical analyses were performed using Python 3.13 with the SciPy (Minneapolis, MN: SciPy Community), NumPy (Austin, TX: NumPy Developers), and Pandas libraries (Austin, TX: The Pandas Development Team).

## Results

Patient characteristics

**Figure 1:**
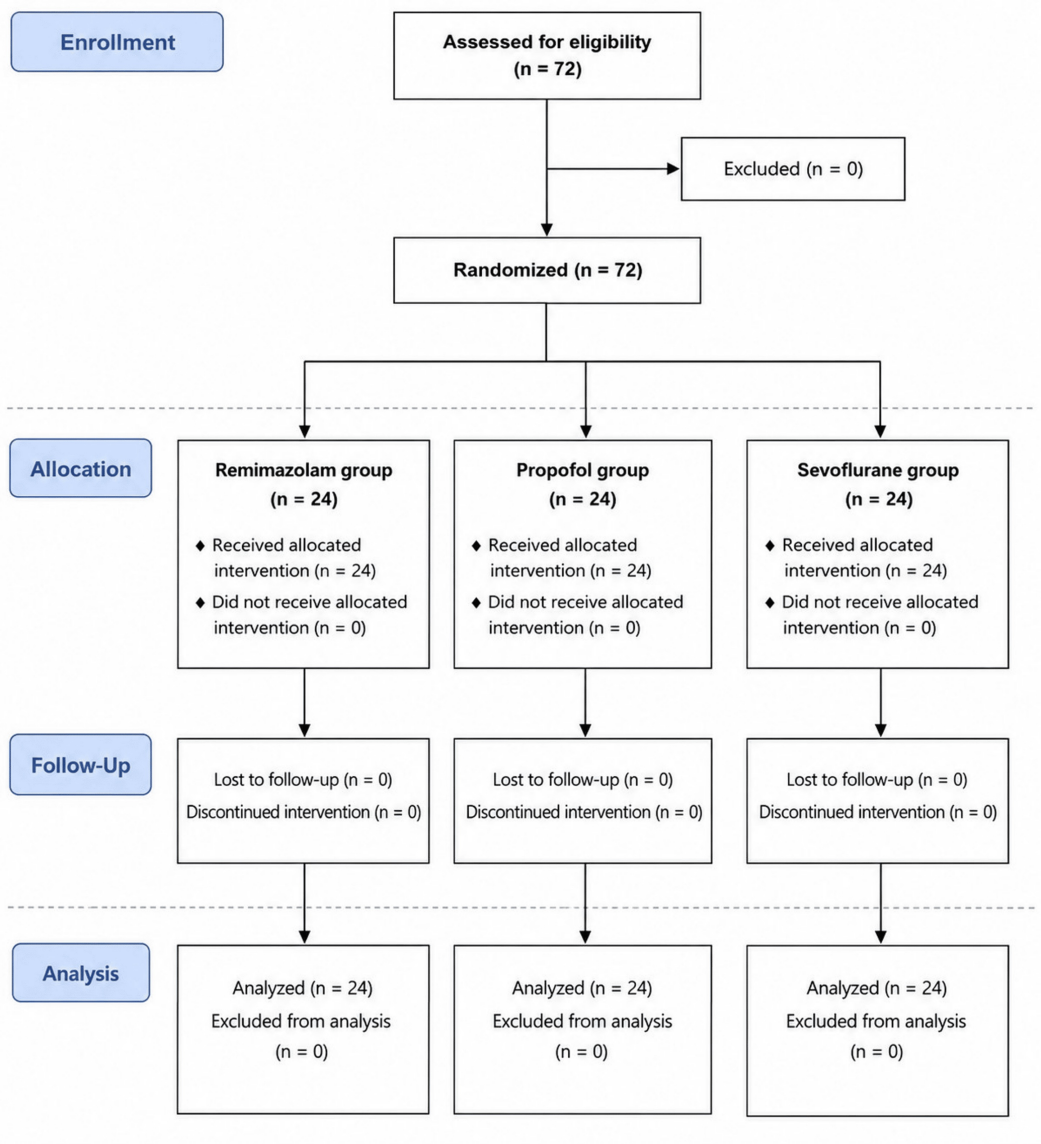
Overview of participant enrollment, group allocation, follow-up, and inclusion in the final analysis.

A total of 72 patients were enrolled, randomized, and allocated equally to the remimazolam, propofol, and sevoflurane groups (24 patients per group). All participants received the assigned anesthetic regimen, completed the study protocol, and were included in the final analysis (Figure 1). Baseline demographic and perioperative characteristics are summarized in Table 1.

**Table 1:** Baseline demographic and perioperative characteristics of the study participants. Values are presented as mean ± standard deviation or number (%). ASA: American Society of Anesthesiologists

Variables	Remimazolam (n = 24)	Propofol (n = 24)	Sevoflurane (n = 24)	p-Value
Age (years), mean ± SD	43.4 ± 6.4	45.5 ± 8.2	44.6 ± 6.0	0.597
Height (cm), mean ± SD	159.3 ± 6.1	158.3 ± 4.6	160.1 ± 4.9	0.493
Weight (kg), mean ± SD	55.9 ± 5.8	56.5 ± 4.1	57.8 ± 5.8	0.445
BMI (kg/m²), mean ± SD	22.0 ± 1.6	22.6 ± 1.9	22.5 ± 2.0	0.477
ASA I, n (%)	6 (25.0)	5 (20.8)	6 (25.0)	0.926
ASA II, n (%)	18 (75.0)	19 (79.2)	18 (75.0)	-
Operation time (min), mean ± SD	73.2 ± 21.2	73.3 ± 21.4	89.0 ± 46.3	0.147
Anesthesia time (min), mean ± SD	120.9 ± 24.2	121.4 ± 25.3	127.5 ± 42.3	0.724

Group comparisons demonstrated no significant differences in age (p = 0.597), height (p = 0.493), weight (p = 0.445), body mass index (p = 0.477), ASA physical status (p = 0.926), operation time (p = 0.147), or anesthesia time (p = 0.724). The mean age was 43.4 ± 6.4 years in the R group, 45.5 ± 8.2 years in the P group, and 44.6 ± 6.0 years in the S group. Mean BMI values were 22.0 ± 1.6 kg/m², 22.6 ± 1.9 kg/m², and 22.5 ± 2.0 kg/m², respectively. The distribution of ASA physical status was comparable among groups, with ASA II patients accounting for 75.0%, 79.2%, and 75.0% of patients in the R, P, and S groups, respectively.

Changes in regional cerebral oxygen saturation

**Figure 2:**
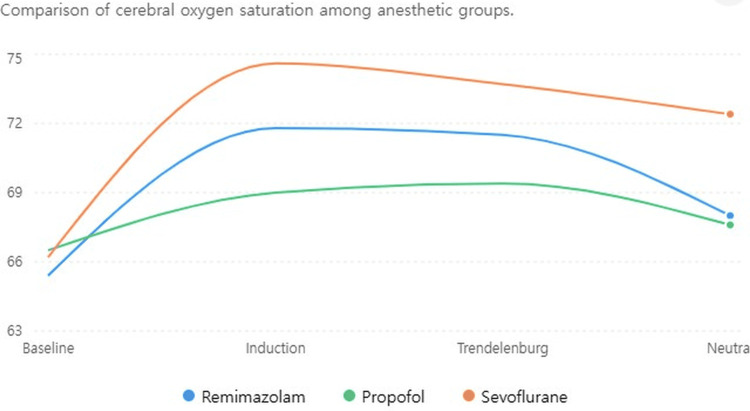
Changes in regional cerebral oxygen saturation over time.

Changes in rSO₂ are summarized in Table 2 and Figure 2. Baseline rSO₂ values were comparable among the three groups (R: 65.8 ± 6.4%, P: 66.5 ± 6.1%, S: 66.1 ± 7.3%; p = 0.944). Repeated-measures ANOVA demonstrated a significant effect of time on rSO₂ (F {3, 207} = 32.89, p < 0.001) and a significant group-by-time interaction (F {6, 207} = 3.58, p = 0.002). However, the overall group effect was not statistically significant (F {2, 69} = 1.71, p = 0.189).

**Table 2:** Changes in regional cerebral oxygen saturation.

Time point	Remimazolam	Propofol	Sevoflurane	p-Value
Baseline, mean ± SD	65.8 ± 6.4	66.5 ± 6.1	66.1 ± 7.3	0.944
Induction, mean ± SD	71.8 ± 8.3	69.0 ± 7.8	74.6 ± 7.2	0.053
Trendelenburg, mean ± SD	72.4 ± 11.1	69.4 ± 8.4	74.1 ± 7.8	0.209
Neutral position, mean ± SD	68.0 ± 8.4	67.6 ± 8.8	72.8 ± 7.2	0.054

After anesthetic induction, rSO₂ increased in all groups. Mean rSO₂ values at induction were 71.8 ± 8.3% in the R group, 69.0 ± 7.8% in the P group, and 74.6 ± 7.2% in the S group. During Trendelenburg positioning, rSO₂ values remained preserved at 72.4 ± 11.1%, 69.4 ± 8.4%, and 74.1 ± 7.8%, respectively. After returning to the neutral position, rSO₂ values were 68.0 ± 8.4%, 67.6 ± 8.8%, and 72.8 ± 7.2%, respectively. No statistically significant differences in rSO₂ were observed among groups at any individual measurement time point.

Bispectral index

**Figure 3:**
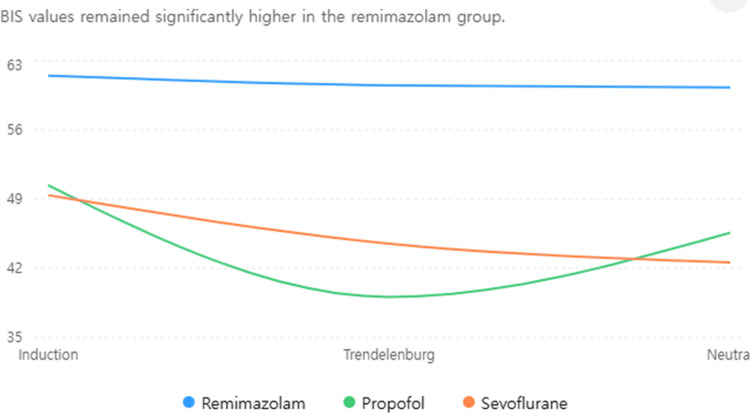
Changes in BIS values over time. BIS: bispectral index

BIS values are presented in Table 3 and Figure 3. BIS values differed significantly among groups throughout the study period. Repeated-measures ANOVA showed significant effects of group (F {2, 69} = 45.81, p < 0.001), time (F {2, 138} = 9.01, p < 0.001), and group-by-time interaction (F {4, 138} = 3.07, p = 0.019).

**Table 3:** Changes in bispectral index.

Time point	Remimazolam	Propofol	Sevoflurane	p-Value
Induction, mean ± SD	61.5 ± 6.7	50.4 ± 16.2	49.4 ± 7.1	<0.001
Trendelenburg, mean ± SD	60.5 ± 5.7	39.1 ± 11.9	44.5 ± 9.5	<0.001
Neutral position, mean ± SD	60.3 ± 6.8	45.6 ± 7.9	42.6 ± 8.7	<0.001

At induction, BIS values were 61.5 ± 6.7 in the R group, 50.4 ± 16.2 in the P group, and 49.4 ± 7.1 in the S group (p < 0.001). During Trendelenburg positioning, BIS values were 60.5 ± 5.7, 39.1 ± 11.9, and 44.5 ± 9.5, respectively (p < 0.001). After returning to the neutral position, BIS values were 60.3 ± 6.8, 45.6 ± 7.9, and 42.6 ± 8.7, respectively (p < 0.001). Post hoc pairwise comparisons with Bonferroni correction demonstrated that BIS values were significantly higher in the remimazolam group than in both the propofol and sevoflurane groups at all study time points (all Bonferroni-adjusted p < 0.05). No significant differences were observed between the propofol and sevoflurane groups.

Hemodynamic variables

**Figure 4:**
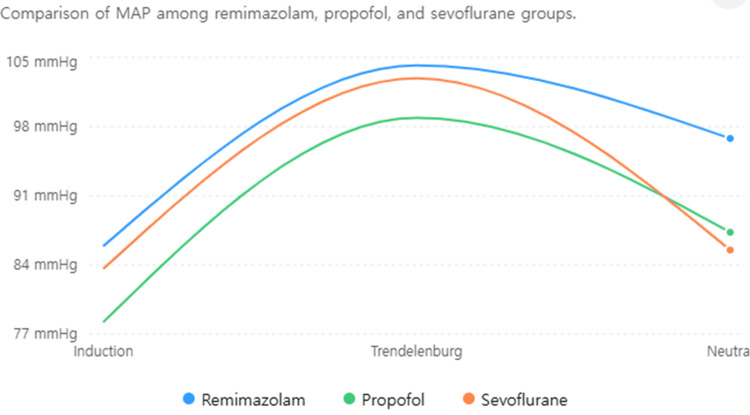
Changes in mean arterial pressure over time. MAP: mean arterial pressure

Mean arterial pressure (MAP) measurements are summarized in Table 4 and Figure 4. Mean arterial pressure (MAP) changed significantly over time (F {2, 138} = 76.81, p < 0.001). A significant group effect was also observed for MAP (F {2, 69} = 3.77, p = 0.028), whereas the group-by-time interaction was not statistically significant (F {4, 138} = 2.19, p = 0.073).

**Table 4:** Changes in mean arterial pressure.

Time point	Remimazolam	Propofol	Sevoflurane	p-Value
Induction, mean ± SD	85.9 ± 13.0	78.2 ± 14.0	83.6 ± 8.5	0.082
Trendelenburg, mean ± SD	104.2 ± 11.3	98.9 ± 12.9	102.9 ± 14.7	0.348
Neutral position, mean ± SD	96.8 ± 13.5	87.3 ± 10.5	85.5 ± 11.5	0.003

At induction, MAP values were 85.9 ± 13.0 mmHg in the R group, 78.2 ± 14.0 mmHg in the P group, and 83.6 ± 8.5 mmHg in the S group (p = 0.082). During Trendelenburg positioning, MAP values were 104.2 ± 11.3 mmHg, 98.9 ± 12.9 mmHg, and 102.9 ± 14.7 mmHg, respectively (p = 0.348). After returning to the neutral position, MAP was significantly higher in the R group than in the P and S groups (96.8 ± 13.5 mmHg, 87.3 ± 10.5 mmHg, and 85.5 ± 11.5 mmHg, respectively; p = 0.003). Post hoc pairwise comparisons with Bonferroni correction demonstrated that mean arterial pressure after return to the neutral position was significantly higher in the remimazolam group than in both the propofol and sevoflurane groups (Bonferroni-adjusted p = 0.030 and p = 0.010, respectively), whereas no significant difference was observed between the propofol and sevoflurane groups.

Heart rate data are summarized in Table 5. Repeated-measures ANOVA demonstrated significant effects of time (F {2, 138} = 24.33, p < 0.001) and group (F {2, 69} = 6.82, p = 0.002), whereas the group-by-time interaction was not significant (F {4, 138} = 1.60, p = 0.177).

**Table 5:** Changes in heart rate.

Time point	Remimazolam	Propofol	Sevoflurane
Induction, mean ± SD	81.7 ± 15.7	74.8 ± 13.6	80.7 ± 16.3
Trendelenburg, mean ± SD	85.5 ± 12.9	72.5 ± 9.9	78.3 ± 12.4
Neutral position, mean ± SD	76.9 ± 11.0	62.9 ± 10.2	71.7 ± 10.2

At induction, HR values were 81.7 ± 15.7 beats/min in the R group, 74.8 ± 13.6 beats/min in the P group, and 80.7 ± 16.3 beats/min in the S group. During Trendelenburg positioning, HR values were 85.5 ± 12.9, 72.5 ± 9.9, and 78.3 ± 12.4 beats/min, respectively. After return to the neutral position, HR values were 76.9 ± 11.0, 62.9 ± 10.2, and 71.7 ± 10.2 beats/min, respectively. Overall, HR tended to be higher in the remimazolam group than in the propofol group throughout the study period.

Cerebral oxygen desaturation and postoperative outcomes

Cerebral oxygen desaturation was defined as a decrease in rSO₂ greater than 20% from baseline or an absolute rSO₂ value below 40. Based on the recorded study time points, cerebral oxygen desaturation occurred in one of 24 patients (4.2%) in the propofol group and in none of the patients in the remimazolam or sevoflurane groups, with no significant difference among groups (p = 0.363). No patient developed persistent cerebral desaturation requiring specific intervention. The incidence of cerebral oxygen desaturation did not differ significantly among the three groups.

No postoperative neurological deficits were observed in any patient. Postoperative nausea and vomiting, shivering, and postoperative pain occurred in a small number of patients and were managed with appropriate rescue medications. No serious adverse events related to cerebral oxygenation were observed.

## Discussion

The present study compared the effects of remimazolam, propofol, and sevoflurane anesthesia on rSO₂ during gynecologic laparoscopic surgery performed in the Trendelenburg position. The principal findings of this study were as follows: first, cerebral oxygen saturation was well preserved throughout the perioperative period in all three groups; second, although the sevoflurane group demonstrated numerically higher rSO₂ values than the remimazolam and propofol groups, no significant overall group effect was observed; third, BIS values were significantly higher in the remimazolam group than in the propofol and sevoflurane groups. Finally, remimazolam was associated with higher mean arterial pressure after return to the neutral position, suggesting better preservation of systemic hemodynamics.

Near-infrared spectroscopy (NIRS) has become widely used for perioperative assessment of regional cerebral oxygenation because it enables continuous monitoring without the need for invasive procedures [10-12]. Unlike conventional hemodynamic parameters, rSO₂ reflects the balance between cerebral oxygen delivery and consumption at the tissue level and may provide early information regarding changes in cerebral oxygen status. Previous studies have reported associations between cerebral oxygen desaturation and unfavorable postoperative outcomes, including delayed recovery and neurocognitive impairment. Furthermore, strategies aimed at preventing substantial reductions in rSO₂ have been associated with improved neurological outcomes in selected surgical populations. Consequently, preservation of adequate cerebral oxygenation remains an important consideration during anesthetic management, particularly in patients who may have limited cerebrovascular reserve.

The combination of carbon dioxide insufflation and Trendelenburg positioning may alter several physiological factors that influence cerebral oxygenation. Increased carbon dioxide levels can promote cerebral vasodilation, whereas positional changes may affect cerebral venous return and intracranial dynamics. As a result, alterations in cerebral perfusion and oxygen balance may occur during laparoscopic procedures. Previous studies examining rSO₂ during Trendelenburg-positioned surgery have produced inconsistent findings, with some investigators reporting decreases in cerebral oxygen saturation and others demonstrating stable or increased values [3,4]. In the present study, rSO₂ remained well maintained throughout the perioperative period in all groups, with a tendency toward mild increases following anesthetic induction and positioning.

A numerical tendency toward higher rSO₂ values was observed in patients receiving sevoflurane; however, the difference did not reach statistical significance. This observation may be related to the distinct cerebrovascular effects of volatile and intravenous anesthetic agents. Previous studies have suggested that volatile anesthetics may preserve cerebral oxygenation by reducing cerebral metabolic demand while maintaining or increasing cerebral blood flow, whereas propofol is generally associated with concurrent reductions in cerebral metabolic activity and cerebral perfusion [5]. Consistent with these physiological differences, earlier investigations conducted during laparoscopic surgery reported relatively higher cerebral oxygen saturation during sevoflurane anesthesia compared with propofol-based anesthesia [6,7]. Although a similar numerical trend was observed in the present study, no significant overall group effect was identified. Therefore, the observed differences in rSO₂ should be interpreted as numerical observations rather than evidence of clinically superior cerebral oxygenation. Taken together, these findings indicate that remimazolam, propofol, and sevoflurane were all effective in maintaining cerebral oxygenation under the study conditions.

Remimazolam is a recently introduced intravenous anesthetic that combines the pharmacologic characteristics of benzodiazepines with rapid metabolic clearance. Previous clinical studies have reported favorable hemodynamic profiles during induction and maintenance of anesthesia when compared with propofol [8,9]. Although its efficacy and safety have been investigated in a variety of surgical settings, information regarding its influence on cerebral oxygenation during procedures associated with altered cerebral physiology remains limited. In the present study, remimazolam maintained cerebral oxygenation at levels comparable to those observed with sevoflurane and propofol. Furthermore, patients receiving remimazolam demonstrated significantly higher MAP values following return to the neutral position. Because cerebral perfusion pressure is influenced by systemic arterial pressure, preservation of blood pressure may contribute to maintenance of cerebral oxygenation during periods of physiological stress. Although no significant difference in rSO₂ was observed among groups, the improved hemodynamic stability associated with remimazolam may become more clinically relevant in patients with impaired cerebrovascular reserve, such as elderly individuals or those with cerebrovascular disease. These findings suggest that remimazolam provides cerebral oxygenation comparable to that of propofol and sevoflurane while offering improved hemodynamic stability under the conditions investigated in this study. These findings are consistent with previous studies demonstrating the clinical importance of maintaining cerebral oxygenation during anesthesia [13].

An interesting finding of the present study was the significantly higher BIS values observed in the remimazolam group despite comparable rSO₂ values. Previous investigations have suggested that BIS monitoring may not accurately reflect hypnotic depth during benzodiazepine-based anesthesia because the BIS algorithm was primarily developed and validated using propofol and volatile anesthetics. Consequently, the higher BIS values observed in the remimazolam group may not necessarily indicate lighter anesthesia but rather reflect limitations of BIS monitoring during benzodiazepine administration. Interestingly, despite significantly higher BIS values, cerebral oxygen saturation remained comparable among the three groups. This observation indicates that the higher BIS values observed during remimazolam anesthesia did not translate into measurable differences in cerebral oxygenation. Preservation of cerebral oxygenation may therefore be influenced more by cerebral perfusion and metabolic demand than by BIS-derived hypnotic depth alone. Consequently, BIS values should be interpreted cautiously during remimazolam anesthesia, particularly when used as the sole indicator of anesthetic depth. This interpretation is further supported by the absence of clinical evidence suggesting inadequate anesthesia or awareness in any patient.

Several limitations of this study should be acknowledged. First, NIRS measures regional oxygenation within the frontal cortex and may not accurately represent global cerebral oxygenation [1]. Second, this study included relatively healthy adults aged 20-65 years and excluded patients with a BMI ≥ 25 kg/m². The BMI criterion was selected to minimize potential confounding effects of obesity on respiratory mechanics, carbon dioxide handling, and cerebral oxygenation during pneumoperitoneum and Trendelenburg positioning. However, these selection criteria may limit the generalizability of the findings to elderly patients, individuals with significant cerebrovascular disease, and overweight or obese patients, who may be more susceptible to cerebral oxygen desaturation. Third, invasive arterial blood pressure monitoring was not performed because arterial cannulation was not routinely used for these procedures. Consequently, noninvasive blood pressure measurements were used to evaluate hemodynamic changes. In addition, arterial blood gas measurements were not routinely obtained, which limited direct assessment of arterial carbon dioxide tension and its potential influence on cerebral oxygenation. Fourth, the number of participants included in this study may have reduced the statistical power to identify modest between-group differences in cerebral oxygenation. Finally, episodes of clinically relevant cerebral oxygen desaturation occurred infrequently, limiting our ability to evaluate potential differences in the incidence of these events among the study groups. In addition, all patients received midazolam as premedication before surgery. Although the same premedication regimen was applied to all study groups, residual benzodiazepine effects may have influenced BIS measurements and could have contributed to the higher BIS values observed in the remimazolam group. Therefore, the BIS findings should be interpreted with appropriate caution.

## Conclusions

This prospective randomized study demonstrated that cerebral oxygenation was well preserved during gynecologic laparoscopic surgery performed in the Trendelenburg position regardless of the anesthetic technique used. Although sevoflurane was associated with numerically higher rSO₂ values, no significant differences in regional cerebral oxygen saturation were identified among patients receiving remimazolam, propofol, or sevoflurane.

Among the three anesthetic techniques, remimazolam demonstrated cerebral oxygenation profiles comparable to those of propofol and sevoflurane and was associated with higher mean arterial pressure following return to the neutral position. Additional studies involving patients with impaired cerebrovascular reserve may help clarify whether the observed hemodynamic advantages of remimazolam are associated with improvements in postoperative neurological outcomes.
